# Alcohol or Gut Microbiota: Who Is the Guilty?

**DOI:** 10.3390/ijms20184568

**Published:** 2019-09-14

**Authors:** Marica Meroni, Miriam Longo, Paola Dongiovanni

**Affiliations:** 1 Department of Pathophysiology and Transplantation, Università degli Studi di Milano, 20122 Milano, Italy; maricameroni11@gmail.com; 2 General Medicine and Metabolic Diseases, Fondazione IRCCS Ca’ Granda Ospedale Maggiore Policlinico, 20122 Milan, Italy; longo.miriam92@gmail.com

**Keywords:** alcoholic liver disease, gut microbiota, gut-liver axis, leaky gut, intestinal permeability, tight junctions, fecal microbiota transplantation, target therapy

## Abstract

Alcoholic liver disease (ALD), a disorder caused by excessive alcohol intake represents a global health care burden. ALD encompasses a broad spectrum of hepatic injuries including asymptomatic steatosis, alcoholic steatohepatitis (ASH), fibrosis, cirrhosis, and hepatocellular carcinoma (HCC). The susceptibility of alcoholic patients to develop ALD is highly variable and its progression to more advanced stages is strongly influenced by several hits (i.e., amount and duration of alcohol abuse). Among them, the intestinal microbiota and its metabolites have been recently identified as paramount in ALD pathophysiology. Ethanol abuse triggers qualitative and quantitative modifications in intestinal flora taxonomic composition, mucosal inflammation, and intestinal barrier derangement. Intestinal hypermeability results in the translocation of viable pathogenic bacteria, Gram-negative microbial products, and pro-inflammatory luminal metabolites into the bloodstream, further corroborating the alcohol-induced liver damage. Thus, the premise of this review is to discuss the beneficial effect of gut microbiota modulation as a novel therapeutic approach in ALD management.

## 1. Introduction

Chronic alcohol consumption represents one of the most common causes of mortality worldwide [1]. According to the World Health Organization (WHO), 2.3 billion people consume alcohol in the world and about 75 million are classified as having alcohol disorders [2]. Alcohol misuse induces damages to multiple end organs, predominantly the liver, the gut, and the brain, thus triggering multi-systemic injuries. In particular, the liver and to a lesser extent the gastrointestinal tract (GIT) are mainly involved in alcohol metabolism, sustaining the greatest degree of tissue injury by heavy drinking. Alcoholic liver disease (ALD) embraces a broad spectrum of hepatic lesions including asymptomatic steatosis, alcoholic steatohepatitis (ASH), fibrosis, cirrhosis, and hepatocellular carcinoma (HCC) [3].

The correlation between alcohol consumption and liver disease is now widely recognized and the majority of individuals (90%) who regularly consume more than 40–60 g/day of alcohol develop steatosis. If the affected individual ceases drinking, steatosis is a reversible condition. However, in 20–40% of dependent drinkers, steatosis may be complicated by inflammation and fibrosis while cirrhosis develops in about 10–20% [4]. HCC annually occurs in 2–3% of alcohol-related cirrhosis [5,6,7] and only half of the patients are eligible for therapeutic treatments (liver transplantation, resection, and percutaneous ablation) [7]. In patients with alcohol use disorders (AUD), ALD and cirrhosis are the leading causes of death [8]. Therefore, there is an urgent need to develop novel therapeutic interventions for patients affected by severe alcoholic hepatitis.

The susceptibility of alcoholic patients to develop ALD is highly variable. The progression of ALD to more advanced conditions is strongly influenced by several issues, i.e., amount and duration of alcohol abuse, age, gender, ethnicity, comorbidities, nutritional status and by environmental, inherited, and epigenetic factors that also cause differences in susceptibility to liver damage [9]. Among these factors, recent evidence has pointed out to the role of gut microbiota and its metabolites in the pathophysiology of ALD. Indeed, alcohol consumption is responsible for substantial modifications of quality and quantity of intestinal flora (condition referred to as dysbiosis), to mucosal alteration and to enhanced gut permeability, resulting in endotoxemia [10]. Specifically, the increased gut permeability due to alcohol abuse leads to higher lipopolysaccharide (LPS) concentration into portal blood flow which bind to Toll-like receptor 4 (TLR4) and activate nuclear factor kappa-light-chain-enhancer of activated B cells (NF-κB) stimulating in turn, pro-inflammatory cytokines release, reactive oxygen species (ROS) production, and oxidative stress. All these events may induce the activation of resident macrophages, the Kupffer cells and hepatic stellate cells (HSCs), perpetuating inflammation and fibrosis in the liver [11,12].

For this reason, this review aimed to focus on the relevance of gut microbiota modulation in the development and progression of ALD and its promising role as potential diagnostic strategy and therapeutic target in the personalized ALD management.

## 2. Insight into the Gut Microbiota

The gut is the largest reservoir of microorganisms in the body. Indeed, the human gastrointestinal lumen is the physiological habitat for a variety of microbes (bacteria, archaea, fungi, yeast, and viruses). It includes tens of trillions of microbial cells belonging to several different species, which is approximately ten-times the number of somatic cells in the human body and they carry more than three million unique genes [13,14]. The majority of bacterial species belongs to the phyla *Firmicutes* (Gram positive) and to *Bacterioidetes* (Gram negative), while the remnants to *Actinobacteria*, *Fusobacteria, Proteobacteria*, and *Verrucomicrobia* [15,16,17]. The GIT and the intestinal flora are in symbiotic relationship. Indeed, the 85% of total bacteria, such as *Lactobacilli* and *Bifidobacteria,* are commensal organisms, whereas the others, such as *Clostridium* and *Fusobacterium*, could potentially become pathogens [18].

The exact role of microbiota remains largely unexplored. However, the presence of microbes in the gut participates in vitamin (i.e., vitamin B) and amino acid synthesis, macromolecule catabolism, energy providing, drug and toxin metabolism, and intestinal barrier preservation. All imbalances or alterations in intestinal flora taxonomic composition and/or function are generally indicated as ‘dysbiosis’ [19]. In particular, alterations of microbiota homeostasis have an impact not only on alcohol-related liver disorders, but they have been also associated with type II diabetes (T2DM) [20], obesity [21,22], inflammatory bowel disease (IBD) [23], irritable bowel syndrome (IBS) [24], celiac disease [25], cancer [26], and many others. Indeed, gut microbiota interacts with the host, avoiding the pathogenic bacteria over-growth, modulating the immune system and the immunological tolerance of mucosal cells, and stimulating the production of pathogen-specific immunoglobulin A (IgA) [27].

The concentration and the composition of bacterial species are different along the entire GIT with inter-individual variation depending on the age, ethnicity, lifestyle, medications, and dietary habits [18]. Alcohol consumption along with the Western diet, enriched in animal fat and sugars, bowel movement frequency, genetics, and disruption of circadian rhythms can predispose to dysbiosis, as demonstrated in a large number of studies in rodents [28,29] and humans [30,31,32,33,34].

The physiological function of the intestinal flora in the production of metabolites (i.e., endotoxins, bile acids, and short chain fatty acids (SCFAs), products of fermentation of unabsorbed food residues, mainly carbohydrates used as energy source for the host) can be assessed using metabolomic, proteomic, and transcriptomic approaches. Indeed, the intestinal, systemic, uric, and fecal concentration of bacterial products potentially reflects the microbiota composition and may be exploited as a diagnostic noninvasive marker. In addition, sequencing of the 16S ribosomal RNA (rRNA) gene through next-generation DNA sequencing is widely used to determine the bacterial community diversity and their relative abundance in mucosa-associated colonic tissue biopsies and in fecal samples. This technique is now also flanked by the more expensive metagenomics shotgun approaches [34].

### 2.1. Preclinical Models of Alcohol-Induced Microbiota Alterations

Alcohol misuse facilitates intestinal bacterial over-growth predominantly in the upper small bowel, both in preclinical and human models [29,30]. Preclinical models are particularly helpful in the understanding of the implications of the ethanol administration on dysbiosis and bacterial over-growth. Alcohol-fed mice for three weeks developed ALD, related to qualitative changes in the intestinal microflora due to small intestinal bacterial over-growth, dysbiosis in the cecum and suppression of gene and protein expression of bactericidal c-type leptins Regenerating islet-derived protein 3 b (Reg3b) and Reg3g in the small intestine. Moreover, even anti-microbial molecules against Gram-positive bacteria contribute to enteric dysbiosis, observed after ethanol consumption [29]. These mice demonstrated an unbalance of bacterial families, displaying a reduction of bacteria from the phylum *Firmicutes* and conversely an enhanced abundance of *Bacterioidetes* and *Verrucomicrobia.* However, after the treatment of ethanol-fed mice with probiotics, Reg3g protein levels were partially restored, rescuing alcoholic steatohepatitis. In another study, Mutlu and colleagues evidenced that rats intragastrically fed alcohol daily for ten weeks displayed an altered colonic mucosa-associated bacterial composition leading to ileal and colonic dysbiosis [28]. These changes support the role of gut microbiota composition in alcohol-induced injuries both in intestine and in the liver, contributing also to alteration of intestinal barrier integrity and release of pro-inflammatory mediators into the portal circulation. Moreover, both *Lactobacillus GG* (*LGG*) or oats prevented the alcohol-induced colonic dysbiosis in this model.

The severity of alcohol-induced liver injury is associated with a specific microbiota signature in ALD patients and the degree of susceptibility to alcohol-induced liver injury is transmissible from patients to mice through fecal microbiota transplantation (FMT). Germ-free mice fed ethanol develop severe liver inflammation, necrosis, and greater intestinal permeability, when transplanted with the gut microbiota isolated from patients with severe alcoholic hepatitis compared to those that received intestinal microbiota from alcoholic subjects without alcoholic hepatitis [35]. According to this notion, the transfer of intestinal flora from alcoholic patients without alcoholic hepatitis ameliorates alcohol-induced liver lesions, demonstrating that intestinal bacteria play a causative role in the disease development. The bacterial species mainly involved in the deleterious effect of severe ALD transmission included altered *Bacteroides* phylum as well as *Bilophila wadsworthia*, *Alistipes*, *Butyricimonas*, *Clostridium*, *Proteus,* and *Escherichia Coli*. Conversely, intestinal microbiota isolated from patients with advanced alcoholic hepatitis displays hampered concentration of *Faecalibacterium prausnitzii*, a microbial species with anti-inflammatory and mucosal protective properties producing butyric acid [36] and of *Akkermansia muciniphila*, that resides in mucin barrier [35]. Importantly, butyric acid is known as a regulator of gluconeogenesis and energy homeostasis [37]. Llopis and coworkers demonstrated, for the first time, that the susceptibility to ALD is reversible through restoring eubiosis, thus paving the way for a personalized medicine [35]. Analyzing the differences in fecal metabolome, the most striking discriminant metabolites were the bile acids (i.e., the antioxidant ursodeoxycholic acid (UDCA) was reduced in alcohol-fed mice). The presence of ALD-associated microbiota was related also to a marked reduction of mucins (predominantly Muc2), Reg3b and Reg3g expressions, contributing to dysbiosis and to increase the amount of the adherent bacteria and to bacterial translocations [29].

Nonetheless, changes in the mycobiome composition, i.e., *Candida spp.* overgrowth, induces also the translocation of β-glucans into systemic circulation and antifungal treatment results in the improvement of alcohol-induced liver injuries [38]. All these events may influence the host susceptibility to ALD.

### 2.2. Impact of Alcohol Consumption on Human Gut Microbiota

Changes in gut microbiota due to the alcohol misuse have been suggested as a key factor in the development of ALD and alcohol-related morbidity and they have been largely explored in several studies [30,31,32,39,40,41,42,43,44,45]. In particular, chronic alcohol overconsumption favors small intestinal malabsorption and modification of colonic bacteria balance, altering also gut microbiota metabolism, as it has been previously demonstrated by Steinar Traae Bjørkhaug and colleagues by using the d-xylose breath test [46]. This approach was extensively used in the past years as a tool to diagnose small intestinal bacterial overgrowth, taking advantage of the microbial ability to metabolize d-xylose and to assess the effective absorption area of the small intestine [46]. In this study, the authors determined that chronic alcohol overconsumption causes malabsorption comparable to what was observed in patients with untreated celiac disease, and that it may alter gut microbial metabolism [46]. This phenomenon is usually described with the term ‘leaky gut’.

More recently, Traae Bjorkhaug and colleagues analyzed the variability in the intestinal bacterial composition in patients with chronic alcohol overconsumption compared to those with no or very low history of alcohol intake [45]. These patients had a potentially more inflammatory active microbiota along with an over-representation of *Proteobacteria* (Gram-negative, increasing endotoxins in the blood) in the gut, at the phylum level, and a particular increase in the genera *Clostridium, Holdemania (Firmicutes)*, and *Sutterella* (*Proteobacteria*). On the contrary, they showed a lower amount of the genus *Faecalibacterium*, as previously demonstrated in preclinical studies, that is generally considered to be protective against gastrointestinal conditions. Moreover, among the assessed SCFA, the authors found a lower concentration of butyric acid, beneficial for the maintenance of the intestinal barrier integrity, in the feces of alcohol over-consumers. Indeed, the composition of intestinal bacteria influences the amount and the type of metabolites produced.

All these results are in line with the previous studies in which lower amounts of *Bacteroidetes* and *Lactobacillus* species and a higher abundance of *Proteobacteria* and *Fusobacteria* in alcoholics have been found [31,32]. To date, the reason for the bacterial overgrowth remain unexplained. However, since alcohol can reduce the gastrointestinal motility, this has been proposed as a pivotal mechanism for fecal stasis and lumen bacterial proliferation. Nonetheless, even the suppression of innate and adaptive immune response could exert a direct effect on intestinal microbiota alterations.

Overall, alcoholics and cirrhotic patients demonstrate a microbial community enriched in *Proteobacteria* of the class *Gammaproteobacteria* and *Firmicute* of the class of *Bacilli*, and the degree of the over-growth correlates with the severity of the liver damage [41]. Furthermore, even the enhanced presence of the pathogenic families of *Enterobacteriaceae* and *Streptococcaceae* and the decreased of *Akkermansia muciniphila* are strictly correlated with the severity of alcoholic hepatitis [35,47]. All these alterations are also accompanied by increasing concentrations of secondary bile acids, paralleling the liver damage worsening [35]. The restoration of the balance of these microorganisms improved alcohol-associated hepatic injury and gut barrier function in a mouse model of ALD [47]. However, specific changes in the class of *Clostridia* may differ between ALD and alcohol-related cirrhosis, possibly identifying the stage of the liver disease.

Finally, a decrease in fungal diversity and an over-representation of *Candida* species in patients with AUD, independently of liver disease stage, has been observed [38].

## 3. Physiological Functions of Gut Barrier

The bidirectional flux between the gut and the liver through the biliary tract, portal vein, and systemic circulation represents a crucial player on liver pathology. In particular, the liver modulates gut microbiota composition, exerting multiple functions (i.e., bile acid production) and participating with the enterohepatic circulation which is accountable for the responsiveness to gut bacterial end-products and nutrients received via the portal vein. The gut-liver also axis has implications for the intestinal immune response, intestinal barrier function, and hepatic and systemic inflammation [36]. The intermediate frontier that separates the gut-liver axis is represented by the intestinal barrier, comprising multiple layers that line the intestine and protect the hosts from microorganism invasion. This barrier is not only a selectively permeable physical barrier constituted by juxtaposed epithelial intestinal cells held together by tight junctions (occludins, claudins, and zonula occludens 1 (ZO-1)), adherent junctions and desmosomes, but also has immunological properties, due to the presence of the mucus edge. The intestinal mucus layer is composed by mucin (predominantly Muc2 in small and large intestine) secreted by goblet cells and acts as a physical, chemical, and immunological barrier, protecting also mucosal cells against exogenous agents. Indeed, it may serve as binding sites for commensal microorganisms on the surface, that are considered a barrier for the colonization of pathogens. Moreover, selected commensal species from different phyla, as the *Akkermansia muciniphila,* exploit the mucin oligosaccharide chains as a carbon source. In germ-free rats, the depletion of microbiota induces an imbalance between mucus production and degradation, leading to a doubling in the thickness of the mucus layer and swelling of the cecum due to the accumulation of mucus, and the resulting retention of water [48]. The excessive erosion of the protective mucus layer, conversely, has been associated with pathogens over-growth.

The inner portion of the mucus, instead, is sterile. In this region, the intestinal epithelial cells and the immune cells (Paneth cells) secrete antimicrobial peptides (for example defensins, lysozyme, and c-lectin Reg3g) and IgA produced by plasma cells, in order to largely devoid the migration of bacteria into the inner mucus layer. Interesting, the majority of IgA produced in the gut are not reactive to the commensal microbiota. The reduction of these mediators is associated with bacterial over-growth in both preclinical and human studies [49]. Intestinal over-expression of Reg3g may prevent the effect of chronic alcohol consumption on liver damage in mice, even in presence of increased intestinal permeability [49].

An additional defense against microorganism invasion is constituted by a lamina propria, which connects the surface of the mucosal epithelium to the basement *muscolaris mucosae*, localized in the sub-epithelial space along with its resident population of innate and adaptative immune cells. It regulates the translocation of antigens and prevents microbial displacement. However, in case of microorganism translocation through the intestinal epithelium to the bloodstream, they are eliminated by lamina propria-resident macrophages or engulfed by dendritic cells and carried to mesenteric lymph nodes [50].

The gut-associated lymphoid tissue (GALT), consisting in isolated or aggregated lymphoid follicles forming Peyer’s patches in small intestine, is also an extremely important component of the intestinal barrier. It contains the majority of the immunoglobulin-secreting cells of the entire body, by hosting naïve immune cells differentiation into a variety of mature immune cell subsets [50].

Finally, even blood vessels, smooth muscle cell layers and components of the enteric nervous system (ENS), guarantee the intestinal barrier function by regulating the mucosa and by initiating specific defense programs in case of microorganism invasions [51].

## 4. Hallmarks of Alcohol Misuse in Intestinal Barrier Integrity

The disruption of the intestinal barrier function is relevant for alcohol-induced liver pathology and its etiology is multifactorial. After two weeks of alcohol administration in rodents, the leakiness of the intestinal barrier is evident, and then, after another two weeks, endotoxemia and hepatic injuries occur [52]. Ethanol and its metabolites may alter gut permeability exerting a direct deleterious effect on the adherence junctions and on tight junction integrity such as ZO-1, as a consequence of increase oxidative stress burden in the intestine [53,54]. It also induces mucus erosions and ulcerations modifying the glycosylation of the protective mucus layer and potentially the abundance and composition of the adherent bacterial species [55].

Furthermore, the alteration in microflora taxonomic composition and dysregulation of bile acid metabolism, that usually occurs in ALD patients, may trigger the dysfunction of barrier properties and enhance intestinal permeability. Therefore, dysbiosis may contribute to intestinal hypermeability, and in particular, the exaggerated presence of *Proteobacteria* may favor an intestinal mucosa inflammation [56]. In addition, microbial metabolites, such as SCFAs (butyrate, acetate, and propionate), that have been reported to be altered after alcohol consumption, may affect the intestinal barrier integrity and mucosal immune tolerance [57]. For example, *Faecalibacterium prausnitzii*, a member of *Firmicutes* phylum that is down-modulated in ALD patients, is an example of microbial species producing butyric acid. The reduction of this microbial product weakens the firm connection between the intestinal epithelial cells, by decreasing the expression of the tight junction proteins and of mucins [58,59]. The restoration of the physiological abundance of microorganisms producing butyrate, in turn, may ameliorate the gut high permeability and systemic inflammation [60]. Finally, the increasing intestinal expression of several microRNAs (miRNAs; i.e., miR-122 and miR-212), short non-protein coding single-strands RNAs involved in the regulation of cell–cell cross-talk, may also affect gut barrier integrity by targeting ZO-1, claudins, and occludins, as shown in both Caco-2 cells and mice enterocytes [9].

The enhanced intestinal permeability facilitates the translocation, from the intestinal lumen to extraintestinal space, of viable pathogenic bacteria, Gram-negative microbial products, and pro-inflammatory luminal metabolites into the blood circulation, contributing to liver damage [28,61]. Lipoteichoic acid, flagellin, bacterial hypomethylated (CpG) DNA, and other gut-derived toxins are released in the blood flow and are implicated in the development of ALD. Among them, endotoxins (LPS) and peptidoglycans are the most representative. When these pathogenic antigens reach the liver via the portal vein, they are enabled to induce the activation of the inflammatory processes [62], becoming responsible not only for the worsening of hepatic injury, but also for mortal infections in cirrhotic patients [62]. The massive exposure of the liver parenchyma to LPS may be relevant for inflammatory and fibrogenic processes perpetuation. Indeed, LPS stimulates the innate immunity response, through the binding to TLR4 and its co-receptor CD14, activating in turn NF-κB and interleukin-6/signal transducer and activator of transcription 3 (IL6/STAT3) signaling in Kupffer cells, macrophages, and HSCs. Moreover, peptidoglycans, components of Gram-positive bacteria, interact with TLR2 on circulating peripheral blood mononuclear cells (PBMCs) thus eliciting inflammatory responses [63]. Similarly, chronic alcohol consumption favors intestinal fungal translocation into systemic circulation, contributing to liver inflammation via C-type lectin-like receptor (CLEC7A) on Kupffer cells and bone marrow derived cells [38]. As a consequence of these interactions, pro-inflammatory mediators are secreted, such as chemokines and cytokines (TNFα and IL1β), free radicals, and leukotrienes, exacerbating the inflammation on the substrate of already damaged hepatocytes by the alcohol consumption. TLR4 deficient mice exhibit less severe liver injury after alcohol administration, supporting the role of endotoxins in liver injury onset [12]. The impairment of intestinal barrier may lead also to bacterial displacement to mesenteric lymph nodes, further corroborating the systemic inflammation [64].

The diagnostic approaches to study the modifications of the intestinal barrier functionality are essential for staging ALD progression. Therefore, several strategies for the assessment of structural impairment of this barrier have been developed [65]. These strategies mainly exploit the analyses of flux rates across the intestinal wall of defined inert molecules, that can be adequately measured in blood and in urine. For instance, the oral administration of the indigestible and radioactive probe 51Cr-EDTA and its dosage in urine is widely used to investigate the permeability of the whole intestine. Conversely, to specifically address the analyses of the small intestine permeability, the ingestion of non-metabolizable oligosaccharides of large size, e.g., lactulose or sugars of small size, e.g., mannitol or l-rhamnose, are generally used [51]. These techniques may be also flanked by the assessment of serum/plasma and fecal biomarkers and bacterial metabolites dosage, and by breath tests, assessing the bacterial compounds exhaled.

**Figure 1:**
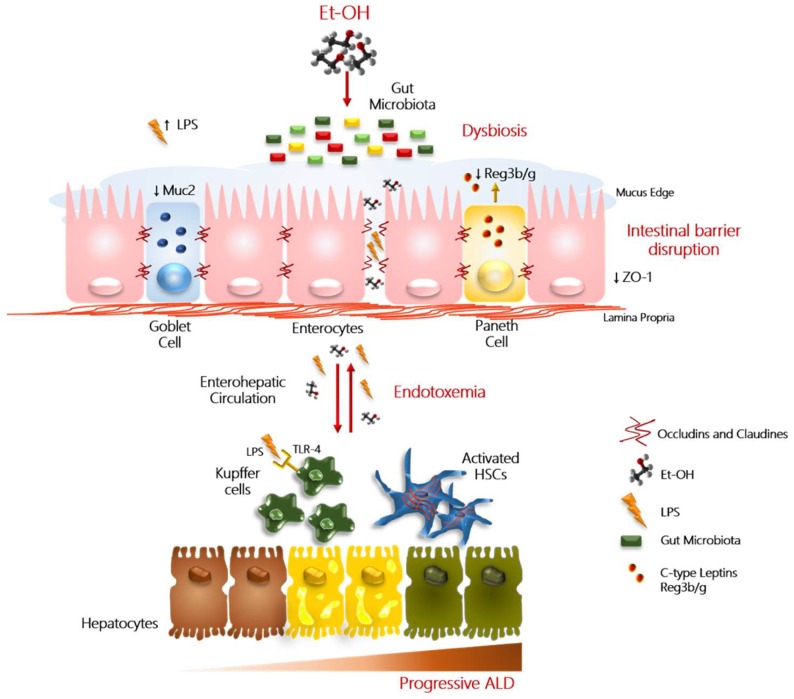
*Role of ‘leaky gut’ in progressive ALD.* In this figure, we reported a schematic illustration of the main alterations that contribute to alcohol-induced liver injury. Excessive alcohol abuse (Et-OH) exerts a direct detrimental effect on the intestinal barrier integrity by affecting the tight junction protein (i.e., zonula occludens 1 (ZO-1)) expression and mucus layer. Physiologically, mucus (Muc2) is produced by the goblet cells, instead, the Paneth cells (immune cells) secrete antimicrobial peptides (i.e., c-lectin Reg3b/g), that contribute to maintain the inter-species bacterial balance. Alteration in quality/quantity of microflora taxonomic composition (referred to as ‘dysbiosis’) may trigger the dysfunction of barrier properties and enhance intestinal permeability. The hyperpermeability facilitates the translocation into the enterohepatic circulation of viable pathogenic bacteria, Gram-negative microbial products, mainly lipopolysaccharides (LPS). LPS, peptidoglycans, and β-glucans bind Toll-like receptor 4 (TLR4)/CD14 complex, TRL2, and C-type lectin-like receptor (CLEC7A), respectively, and mediate the activation of hepatic Kupffer cells and peripheral blood mononuclear cells (PBMCs), via NF-κB and IL6/STAT3 signaling. Activated Kupffer cells and PBMCs, in turn, release a large amount of pro-inflammatory cytokines and chemokines (such as TNFα and IL1β), further perpetuating the hepatic inflammation and oxidative stress in the context of already damaged hepatocytes. These events can even contribute to the worsening of hepatic injury to advance stages, favoring also the activation of hepatic stellate cells (HSCs), involved in fibrogenic processes.

The impact of alcohol overconsumption on intestinal barrier integrity disruption is schematically represented in Figure 1.

### Impact of Bile Acids on Intestinal Barrier Integrity

Emerging evidence has suggested that alterations in bile acids metabolism are associated with alcohol-related disorders and hepatic comorbidities, i.e., development of cholestasis. In the physiological status, the liver synthetizes bile acids and releases them into the duodenum lumen as conjugated metabolites which participate in enterohepatic cycling upon gut microbial modifications. In particular, bile acid activation of intestinal farnesoid X receptor (FXR) leads to the transcription of fibroblast growth factor 19 (FGF19), which in turn modulates gut barrier integrity and downregulates bile acids synthesis in the liver [66]. Alcohol consumption reduces FXR expression as well as stimulates cholesterol 7-α hydroxylase 1 (Cyp7A1) hepatic activity, resulting in higher bile acid pool in the liver. Indeed, actively drinking patients with cirrhosis increased fecal secondary bile acids (deoxycholic acid (DCA) and lithocholic acids) compared to healthy control or abstinent cirrhotic individuals [67]. Furthermore, higher levels of DCA may prevent the growth of beneficial members of *Bacteroidetes* and *Firmicutes* phyla, leading to HSCs activation and increased risk of HCC development [68].

## 5. Gut Microbiota: The Mirror of your Addiction

Recent evidence suggests that modifications in the composition of gut microbiota and its metabolites may influence the brain functions and behavioral aspects [69]. In particular, alcohol-induced enhanced intestinal permeability and endotoxemia may affect phycological status and cognitive ability, reinforcing the drinking tendency. The proof of this concept has been provided by Leclercq and colleagues who showed that in noncirrhotic patients hospitalized for alcohol detoxification, the increasing intestinal permeability and LPS serum dosage were significantly correlated with higher scores of depression, anxiety, decreased social interactions, and ethanol cravings, even after alcohol withdrawal [70]. The ‘leaky gut’, indeed, may alter central nervous system (CNS) functionality. Nevertheless, in ALD-cirrhotic patients, systemic inflammation, endotoxemia, and hyperammonemia are strictly connected with the manifestation of hepatic encephalopathy [71]. The systemic inflammation induces a detrimental direct effect on neurons and favors the disruption of the blood-brain barrier (BBB) and the passage of activated peripheral immune cells, thus enhancing the ethanol-induced neuroinflammation mainly in microglia and astrocytes [72]. Microbial metabolic products may also have a strong impact on peripheral inflammation and brain barrier integrity. Gut bacteria produce neurotransmitters (such as serotonin, gamma-aminobutyric acid (GABA), dopamine, acetylcholine), and bacterial fermentation of dietary fiber induces the release of SCFAs with potential neuroactive properties [64]. For instance, butyric acid mainly derived from *Firmicutes*, may influence the immune milieu of brain through changing peripheral immune system function and attenuating neuroinflammation. In addition, it regulates serotonin and gut hormone levels in the enteric nervous system [73]. Serotonin is a neurotransmitter that can modulate intestinal motility through its 5-HT receptors on different cell types of the GIT, mainly enterocytes, enteric neurons, and immune cells and, given its role in humor regulation, the effect of microbiota composition on social behavior could be explained.

Several studies support the notion that antimicrobials, probiotics, and high fiber butyrate-producing diets may positively influence the health of the brain, ameliorating memory and cognition [74].

## 6. Promising Therapeutics to Modulate Gut Microbiota in ALD

Current standards of care for the management of ALD patients aim to prevent either liver damage onset or progression by encouraging alcohol abstinence. However, the efficacy of treatments is highly dependent from disease severity and the ability to overcome alcohol withdrawal with appropriate psychological and pharmacological approaches, which has success rates between 20–40% of remission [75,76]. The global burden of alcohol-related deaths and disabilities is estimated to be 3.8% and 4.6%, respectively, and most of these are due to alcoholic cirrhosis and liver failure [77,78]. To date, liver transplantation represents a potential lifesaving indication for the end-stage of liver disease in ALD candidates. However, this topic is much-debated from both medical and ethical grounds as it is regarded as a “self-inflicted disease” and also due to the ever-increasing demand of donor organs combined with alcohol relapse and cravings [78]. Regardless the stage of disease, alcohol abstinence is considered the cornerstone of therapy as it improves hepatic histology and survival of ALD patients [79]. Nevertheless, around 5–10% of ALD patients are at risk to develop fibrosis and cirrhosis even if they cease drinking [80]. Thus, there is an urgent need to develop novel therapeutic interventions.

Several pre-clinical and human studies strive to explore how alcohol-induced gut microbiota alterations may contribute to liver disease progression whose mechanism remains still unclear. Otherwise, most of them showed an association between ethanol-induced microbiota unbalancing in the GIT, bacterial overgrowth and development of liver pathology. Therefore, intensive efforts have been directed to investigate potential therapeutic strategies aimed to modulate intestinal bacteria composition through 1) untargeted approaches, including diet, probiotics, prebiotics, antibiotics, fecal microbiota transplant (FMT) or 2) precision medicine by selectively targeting microbial and host metabolites [81].

### 6.1. Effects of Diet on Gut Microbiota Communities

Research in animal models and clinical studies have widely demonstrated that the composition of gut microflora may be influenced by genetic, lifestyle, and environmental factors, including dietary intake, which further account for the inter-individual variability of the intestinal bacterial strains. Specific diets, such as high-fat diet (HFD) and Western diet, have been associated with the overgrowth of pro-inflammatory bacterial species, affecting intestinal pH, gut barrier integrity, and favoring LPS transition into the blood flow [82]. These alterations may promote the predisposition to gut microbiota-related disorders, thus paving the way to the study of the effects of diet to reverse alcohol-associated dysbiosis [83,84,85,86].

Both long and short-term dietary changes can rapidly normalize intestinal microflora, thus representing a simple and effective approach to restore eubiosis. The major differences in microflora composition have been observed between people consuming Western diet and subjects with high-fiber dietary habits. An interesting finding has been carried out on American volunteers, who were randomized to receive an animal-based diet (meats, eggs, and cheese) or a plant-based diet (cereals, legumes, fruits, and vegetables). It showed an increment of bile-tolerant species (*Alistipes, Bilophila*, and *Bacteroides*) and reduced levels of *Roseburia, Eubacterium rectale,* and *Ruminococcus bromii* which metabolized dietary plant polysaccharides in individuals under an animal-based regimen [68,87].

Dietary fibers, encompassing complex carbohydrates and oligosaccharides, exert great health benefits as a result of intestinal fermentative metabolism mediated by colonic bacteria that produce SCFAs. Zymmer et al. analyzed the fecal samples of vegan and vegetarian individuals and found a significant reduction of *Bidifobacterium* and *Bacteroides* species similarly to what previously observed by Aries et al. who compared English subjects consuming a mixed Western diet to Africans feeding high-carbohydrate vegetarian diet [88,89]. Also, diet-enriched in carbohydrates and fibers was responsible for lower stool pH (between 5.5 and 6.5) probably due to the end-products of gut fermentative metabolism thereby inhibiting pathogenic bacteria overgrowth like *Escherichia coli* and *Enterobacteriacee* [82,88,90,91]. Long-term fibers consumption is further associated with a prevalence of *Prevotella*, whose levels are generally diminished with animal-based diet. However, certain species belonging to the phyla of *Bacteroidetes*, such as *Prevotella copri* and *Bacteroides vulgatus* have been associated with insulin resistance. Conversely, members of *Verrucomicrobiaceae* and *Ruminococcaceae*, like *Akkermansia muciniphila* and *Faecalibacterium prausnitzii*, respectively, which improve insulin sensitivity, decreased in different animal models fed with HFD [92,93,94,95,96] and alcohol administration [36,97].

Finally, many natural extracts have been found to have hepatoprotective impact on ALD. Dietary polyphenols provided by coffee, green tea, and chocolate have shown beneficial effects by directly interacting with gut microbial communities. Grape polyphenols attenuated the effects of HFD–induced metabolic syndrome (MetS) in C57Bl/6 mice, improving glucose intolerance, levels of serum inflammatory markers, and intestinal barrier integrity. Furthermore, *Akkermansia muciniphila* was dramatically increased while a lower Firmicutes to Bacteroidetes ratio was observed [96]. Flaxseed oil improves inflammatory responses and stimulates *Firmicutes* and *Parabacteriodes* overgrowth in ALD mice [98]. Rhubarb and *Litchi chinensis Sonn.* phenol extracts promote eubiosis, restore intestinal barrier dysfunction and protect from liver inflammation in animal models of binge drinking [97,99]. The benefits of diet-enriched in phenols have been related to the amelioration of alcohol-induced hepatic damage [97] and even more to lower risk of hospitalization in cirrhotic patients [100].

### 6.2. Nutritional Strategies Affecting Intestinal Bacterial Species: Probiotics, Prebiotics, and Synbiotics

Nowadays, the value of the caloric intake overcomes its basic role to provide the necessary nutrients for daily energy demand, but it is acquiring particular attention to prevent a wide spectrum of diseases, i.e., cardiovascular diseases, obesity, allergies, and cancer. Extensive research has been focused on the beneficial effects of probiotics, prebiotics, or synbiotics consumption.

The WHO defines probiotics as “life microorganisms which—when administered in adequate amounts—confer a health benefit on the host” [101]. The most representatives are *Lactobacillus* and *Bifidobacterium* that promote anti-inflammatory environment by enhancing intestinal epithelium growth and survival as well as they may counteract the pathogenic bacteria by producing anti-microbial agents and modulating immune system and host defense [102,103,104]. For instance, *Lactobacillus reuteri* strains formed biofilms which dampen TNFα production by LPS-activated monocytic cells [103], whilst *Lactobacillus salivarius* and *Bifidobacterium infantis* attenuate intestinal inflammation reducing the pro-inflammatory production of Th1-type cytokines [105]. *Lactobacillus rhamnosusGG* (*LGG)* was the first probiotic tested in a rodent model of ALD and it was effective to improve ‘leaky gut’ and hepatic inflammation either alone or enriched with oat fibers [106]. Administration of *LGG* showed protective effects on ethanol-induced liver injury, circulating liver enzymes, such as alanine aminostransferase (ALT) and aspartate aminostransferase (AST), and endotoxemia in Wistar rats [107]. Likewise, the short-term oral treatment with *Bifidobacterium bifidum* and *Lactobacillus plantarum 8PA3* in a randomized study encompassing 66 alcoholic patients was associated with microflora renewal and significantly lowered AST and ALT levels compared to the standard therapy [108]. An open-label study carried out on 12 alcoholic cirrhotic subjects receiving *Lactobacillus casei Shirota* 3 times daily for 4 weeks showed that probiotic consumption restored neutrophil phagocytosis possibly by down-regulating interleukin (IL) 10 secretion and TLR4 expression [109]. Recently, it has been demonstrated that mice feeding Lieber-DeCarli diet supplemented with *Akkermansia muciniphila* were protected against acute and chronic ALD as proven by reduced expression of pro-inflammatory cytokines, systemic endotoxins, and restoration of ethanol-induced intestinal barrier dysfunction [110]. Probiotic VSL#3, a mixture of 8 probiotic strains (mainly *Lactobacillus* and *Bifidobacterium*), prevented changes in tight junction protein expression thus protecting the intestinal barrier function and limited endotoxins transition from the gut lumen to the portal circulation in a rat model of acute ALD [111]. Interestingly, Loguercio and co-workers evaluated the effects of a fixed therapy with probiotic VSL#3 in patients affected by different chronic liver diseases, including 20 ALD patients. Similarly, to pre-clinical studies, they observed improvement in circulating cytokines and oxidative/nitrosative stress parameters and even more in liver pathology [112].

Among nutraceutical strategies used to modify gut microbiota composition, even prebiotics revealed appealing molecules which can attenuate hepatic lesions in both animal models and humans. Prebiotics are non-digestible fibers helpful as substrates for *Lactobacillus* and *Bifidobacteria* proliferation [113] and also they should present several features, including: 1) resistance to acids, bile salts, and hydrolyzing enzymes in the stomach; 2) reaching the large intestine without being absorbed in the upper GIT; and, finally 3) being easily metabolized by nonpathogenic flora [113,114]. The group of prebiotics predominantly tested includes fructo-o-oligosaccharides (FOS), galacto-oligosaccharides (GOS), arabinoxylan-oligosaccharides, inulin, and prebiotics from natural sources (chicory and yacon roots, etc). Over the past years, few studies investigated the prebiotic efficacy in alcoholic models, while no clinical trials have been reported in ALD patients. Inulin, FOS, and GOS consumption increase the abundance of *Bifidobacterium adolescentis*, *Bifidobacterium catenulanum, Lactobacillus spp,* and *Faecalibacterium prausnitzii* while they lower levels of *Bacteroides spp* and *Clostridium spp* with supplement-containing fructans [82,115,116,117]. Ethanol diet-enriched in inulin provided beneficial effects on gut bacteria composition and hepatic injury through raising the amount of *Allobaculum*, *Lactobacillus*, and *Lactococcus* species and suppressing the LPS-activated TLR4 signaling on the hepatic macrophages (Mψ) in C57BL/6J mice [118]. Additionally, Yan and collaborators analyzed the transcriptome in a mouse model of continuous intragastric ethanol infusion, revealing that chronic alcohol assumption down-regulated the expression of Reg3b and Reg3g, whose levels were retrieved upon FOS administration [29].

Finally, synbiotics are evolving as new therapeutics to manage gastrointestinal, metabolic and liver diseases. The term “synbiotic” refers to as synergistic mechanism as combines probiotics with prebiotic compounds, which selectively stimulate probiotic bacteria survival enhancing their beneficial effects. FloraGuard^®^, a synbiotic powder enriched in prebiotics and *Lactobacillus acidophilus*, *Lactobacillus bulgaricus*, *Bifidobacterium bifidum*, *Bifidobacterium longum*, and *Streptococcus thermophilus*, added to an ethanol diet ameliorated hepatic steatosis and inflammation but also improved gut permeability in Wistar rats [119]. In a very recent pre-clinical study, C57BL/6 mice chronically exposed to binge ethanol feeding were protected against hepatic steatosis and liver injury whether they were supplemented with *Faecalibacterium prausnitzii* and potato starch, probably due to increased levels of butyrate and propionate transport into the hepatocytes [120]. Unfortunately, further clinical investigations are required to look into their efficacy for ALD prevention and treatment.

### 6.3. Antibiotics

The long-term administration of antibiotics in therapy is controversial due to side-effects which may occur in patients (i.e., antibiotic resistance, hepatotoxicity, disruption of the gut ecosystem, etc.) [121,122]. However, several experimental models revealed that endotoxemia and alcoholic hepatitis were prevented with antibiotics prophylaxis [123,124,125]. Several classes appear eligible to counteract Gram-negative microbial overgrowth in humans. In particular, rifaximin has been approved for alcohol-related cirrhosis as a preventive strategy of hepatic encephalopathy. It is a nonabsorbable derivative of rifampicin, with broad-spectrum of anti-microbial activities by binding to the β-subunit of bacterial DNA-dependent RNA polymerase [126]. In 2012, the phase 2 interventional non-randomized clinical trial (NCT01069133) on rifaximin administration was completed. It has tested the ability of rifaximin therapy to ameliorate the brain functionality in 20 cirrhotic patients with subclinical form of hepatic encephalopathy. Rifaximin usage was significantly associated with the improvement in cognitive performance and in endotoxemia, showing a shift from pathogenic to beneficial metabolites [127].

### 6.4. Fecal Microbiota Transplantation (FMT): An Uncharted Territory

Fecal microbiota transplantation (FMT), defined as the transfer of stool matter from a healthy donor into the intestinal tract of a recipient, is currently representing an ongoing attractive avenue for gut microbiota editing, although it requires further investigation due to several concerns about safety and potential infectivity. However, encouraging results have been observed in patients with IBD or chronic hepatopathies. In 1958, Eiseman et al. published the first successful FMT in patients with severe pseudomembranous colitis (PMC), even though it was not known that *Clostridium difficile* was the leading cause of developing PMC. Currently, FMT has become a lifesaving procedure for recurrent *Clostridium difficile* infection [128].

Regardless the routes of FMT administration (i.e., capsules, nasogastric /nasojejunal/ endoscopic tubing, etc.), the mechanism by which it may confer health benefits include changes in bacterial species, favoring nonpathogenic microbes’ overgrowth which, in turn, produce antimicrobial agents (bacteriocins) [129]. Interestingly, FMT from healthy donors to a mouse model of chronic ethanol exposure significantly decreased alcohol-related anxiety and depression thus suggesting gut microbiota as a potential target to treat alcohol addiction [130]. Other pre-clinical artwork underlined the promising role of FMT to modulate gut microbiota in models of ulcerative colitis (UC) as well as in both gastrointestinal and non-gastrointestinal diseases [131,132,133,134]. In a recent pilot study, Philips et al. showed that FMT improved gut dysbiosis and clinical outcomes of steroid-ineligible patients with severe alcoholic hepatitis. Moreover, they observed changes in relative abundance of pathogenic (*Klebsiella pneumonia*) and nonpathogenic species (*Enterococcus villorum*, *Bifidobacterium longum*, and *Megasphaera elsdenii*), suggesting that donor microbiota do not replace but rather modify the recipient flora as a proof-of-concept [135]. Nonetheless, the potential therapeutic of FMT in ALD need to be better elucidated.

### 6.5. Microbiota-Targeted Therapy: The New Frontier of Medicine

Nutritional strategies, antibiotics, and FMT have been shown to provide advantages to human health despite exerting broad-spectrum, non-specific effects in modulating intestinal microbiota and byproducts. Thus, new challenges in medicine are gambling on microbiota-targeted therapies (i.e., restoring microbial metabolites, bio-engineered bacteria, and modulation of microbiota pathways) for the clinical diagnosis and prevention of several diseases [36]. These approaches require an in-depth knowledge of metagenomics, host-microbiota interactions, and properties of microbial communities.

Excessive alcohol consumption leads to reduced intestinal butyrate, whose levels along with gut injury and inflammatory markers of liver disease were recovered with prophylactic tributyrin supplementation in a mouse model of ethanol exposure [58,59,60]. Metagenomic and targeted metabolome analysis underlined saturated long-chain fatty acids (LCFAs), which are metabolized by *Lactobacilli* to promote their growth, and bile acid intermediates as potential therapeutics in chronic-binge alcohol models. As expected, the abundance of *Lactobacilli* correlated to LCFAs levels in fecal samples of alcoholic patients and mice feeding an ethanol diet showed a down-regulation in LCFAs synthesis. Furthermore, LCFAs added to alcohol administration foster *Lactobacillus* proliferation and improved hepatic damage in these mice [136]. Additionally, treatment with fexaramine, a FXR agonist, or overexpression of FGF19 normalized intestinal barrier and ethanol-induced liver disease in mice [137].

Finally, bio-engineered bacteria have been designed to precisely counteract pathogen-induced dysbiosis by secreting beneficial molecules in the GIT. *Escherichia coli Nissle 1917* (*EcN*) modified to produce pyrroloquinoline quinone (PQQ) ameliorated liver injury in a rat model of acute alcohol consumption [138]. Hendrikx et al. have recently transformed *Lactobacillus reuteri* to produce IL22 which stimulates the intestinal expression of Reg3g. In both binge drinking mice and humans, ethanol reduced several tryptophan metabolites that, normally, induce the expression of IL22 in innate lymphoid cells type 3 (ILC3). Therefore, mice receiving bio-engineered *Lactobacillus reuteri* restored levels of both IL22 and Reg3g and further improved alcoholic steatohepatitis [139].

## 7. Focus on Ongoing Clinical Trials

The restoration of microbial composition, the modulation of bacterial metabolites and FMT from healthy individuals may represent the subjects of larger and long-term clinical trials. Antibiotics are currently used in clinical care to protect cirrhotic patients against complications, such as spontaneous bacterial peritonitis and hepatic encephalopathy. To date, the use of rifaximin as a possible antifibrotic drug which modulates human gut microbiota is under exploration in a biopsy-controlled, double-blind study (EudraCT, 2014-001856-51) [140]. The dietary fiber usage (inulin supplementation) in ALD patients is under definition even in the interventional study NCT03803709. The normalization of alcohol-related dysbiotic signatures with anti-fibrotic purpose is also the topic of a randomized study, which is based on the administration of Profermin Plus^®^, composed by fermented oats, *Lactobacillus Plantarum 299v*, barley malt, and lecithin, to ALD patients (NCT03863730). Phase I of a randomized, single-blind study is currently recruiting patients with alcohol-induced advanced fibrosis with the aim to ameliorate the inflammation and to improve the prognosis of these patients, after dysbiosis resetting by exploiting FMT (NCT03416751). Furthermore, several randomized control clinical trials point out FMT as a beneficial and safe therapeutic approach in severe ALD, fibrosis, and hepatic encephalopathy (NCT04014413, NCT02862249, NCT02424175, NCT02400216, NCT02496390, NCT01968382, NCT03091010, NCT03827772) [36]. Finally, even the exploration of gut–brain axis in alcohol cue-induced craving is under examination in the observational clinical trial NCT03152760. A schematic description of the main ongoing clinical trials is represented in Table 1.

## 8. Concluding Remarks

Through different routes, gut microbiota composition and function are strongly entangled in the pathogenesis and the progression of liver injury in patients who misuse alcohol. In particular, alcohol-induced dysbiosis along with several other issues concur to increase individual susceptibility to ALD. To date, clinical guidelines indicate alcohol abstinence, corticosteroids, or pentoxifylline, or surgical interventions as a gold standard in the treatment of ALD. Therefore, there is an urgent need to identify novel therapeutic strategies to tailor ALD management. Several studies and clinical trials have suggested that intestinal microflora modulation by exploiting probiotics, prebiotics, synbiotics, or FMT will represent a turning point in the care of ALD patients. Trustworthily, addressing gut microbiota as a new frontier of future medicine will brighten the avenue of personalized interventions, as well, act as a lighthouse in the stormy sea.

Nevertheless, microbiota composition investigation could become an appealing candidate even for diagnosis, attempting to stage liver disease. However, further studies are essential to completely draft the true causality between changes observed in the context of alcohol misuse and hepatic morbidities as well as to pinpoint the mechanisms through which microbiota alterations affect liver pathology in ALD patients. Up to now, the enigma remains to be unraveled: Who is the guilty?

## Figures and Tables

**Table 1:** Clinical trials currently underway addressing the therapeutic modulation of gut microbiota in ALD patients.

Clinical Trial Start-End Date	Status	Study Type	Interventions	Conditions	Objectives	Locations
EudraCT, 2014–001856-51 08/14-ongoing	Recruiting (*n* = 136) *	Interventional Randomized	550 mg of rifaximin twice daily for 18 months vs placebo	Biopsy-verified alcoholic fibrosis	Anti-fibrotic and molecular aspects of rifaximin in ALD	Odense University Hospital (Odense, Denmark)
NCT03803709 07/17–07/22	Recruiting (*n* = 40) *	Interventional Randomized	Dietary Supplement: inulin vs. placebo	Alcoholism	Restore a nutritional balance via a dietary fiber intake And study the intestinal and psychological well-being related to a fiber intake	Université catholique de Louvain Brussels, Belgium
NCT03863730 04/19–02/31	Recruiting (*n* = 40) *	Interventional Randomized	Dietary Supplement: Profermin Plus, vs. Fresubin, dietary supplement	Advanced ALD	Demonstrate that the alcohol-related dysbiosis can be reversed improving the disease progression by modulating microbiota with probiotics for 24 weeks.	FLASH—Centre of Liver Research Odense, Fyn, Denmark Odense University Hospital Odense, Denmark
NCT04014413 07/19–10/24	Not yet recruiting (*n* = 450) *	Interventional Non-Randomized	FMT	ASH, IBS, T2DM, MetS	Investigate the efficacy and safety of FMT in a variety of dysbiosis-associated disorder	The Chinese University of Hong Kong Hong Kong, Shatin, Hong Kong
NCT02862249 03/18–08/20	Recruiting (*n* = 32) *	Interventional Randomized (Phase 3)	FMT vs. placebo	ALD abstinent from alcohol for a minimum of 6 weeks	Assess whether restoring gut microbiota with FMT in patients with advanced cirrhosis is both feasible and safe	King’s College Hospital NHS Foundation Trust Recruiting London, United Kingdom
NCT03091010 04/17–04/19	Recruiting (*n* = 130) *	Interventional Randomized	FMT vs. steroids	Severe Alcoholic Hepatitis	Compare FMT and steroid therapy in patients with Severe Alcoholic Hepatitis	Institute of Liver and Biliary Sciences New Delhi, Delhi, India
NCT03827772 01/19–12/19	Recruiting (*n* = 40) *	Interventional Non-Randomized	FMT vs. standard of care treatment	Severe Alcoholic Hepatitis	Evaluate the role of FMT on short term survival and improvement in scores of prognostic significances (CTP, MELD, MELDNa, mDF) in patients with Severe Alcoholic Hepatitis	Postgraduate Institute of Medical Education and Research Chandigarh, India
NCT03416751 01/18–12/19	Recruiting (*n* = 20) *	Interventional Randomized (Phase 1)	FMT vs. placebo	Cirrhosis Alcohol Abuse	Demonstrate that FMT ameliorates inflammation and prognosis of ALD cirrhotic patients	Hunter Holmes McGuire VA Medical Center Richmond, Virginia, United States
NCT03152760 08/17–12/20	Recruiting (*n* = 69) *	Observational Case-control	NA	Alcoholism	Observe the changes in gut bacteria that may occur in people with AUD	National Institutes of Health Clinical Center Bethesda, Maryland, United States

* Estimated number of participants; NA, not applicable.

## Abbreviations

ALD	Alcoholic Liver Disease
ALT	Alanine Aminostransferase
ASH	Alcoholic Steatohepatitis
AST	Aspartate Aminostransferase
AUD	Alcohol Use Disorder
BBB	Blood-Brain Barrier
CNS	Central Nervous System
CLEC7A	C-type lectin-like receptor
Cyp7A1	Cholesterol 7-α hydroxylase 1
DCA	Deoxycholic Acid
EcN	Escherichia coli Nissle 1917
ENS	Enteric Nervous System
Et-OH	Ethanol
FGF19	Fibroblast Growth Factor 19
FMT	Fecal Microbiota Transplantation
FOS	Fructo-o-oligosaccharides
FXR	Farnesoid X Receptor
GABA	Gamma-aminobutyric acid GABA
GALT	Gut-associated lymphoid tissue
GIT	Gastrointestinal Tract
GOS	Galacto-oligosaccharides
HCC	Hepatocellular Carcinoma
HFD	High Fat Diet
HSCs	Hepatic Stellate Cells
IBD	Inflammatory Bowel Disease
IBS	Irritable Bowel Syndrome
IgA	Immunoglobulin A
IL1β	Interleukin-1 Beta
IL10	Interleukin 10
IL22	Interleukin 22
IL6/STAT3	Interleukin-6/ Signal Transducer and Activator of Transcription 3
ILC3	Innate Lymphoid Cells Type 3
LCFA	Long-Chain Fatty Acids
LGG	*Lactobacillus rhamnosus GG*
LPS	Lipopolysaccharides
MetS	Metabolic Syndrome
miRNAs	microRNAs
Mψ	Hepatic Macrophages
NF-κB	Nuclear Factor Kappa-Light-Chain-Enhancer of Activated B Cells
PMBCs	Peripheral blood mononuclear cells
PMC	Pseudomembranous Colitis
PQQ	Pyrroloquinoline Quinone
Reg3b	Regenerating islet-derived protein 3 b
Reg3g	Regenerating islet-derived protein 3 g
ROS	Reactive Oxygen Species
SCFA	Short Chain Fatty Acid
T2DM	Type 2 Diabetes Mellitus
TLR4	Toll-like receptor 4
TNFα	Tumor Necrosis Factor alpha
UC	Ulcerative colitis
UDCA	Ursodexycholic acid
WHO	World Health Organization
ZO-1	Zonula Occludens-1
